# Histone Variants and Their Chaperones in Hematological Malignancies

**DOI:** 10.1097/HS9.0000000000000927

**Published:** 2023-07-11

**Authors:** Ecem Kirkiz, Oliver Meers, Florian Grebien, Marcus Buschbeck

**Affiliations:** 1 Institute for Medical Biochemistry, University of Veterinary Medicine, Vienna, Austria; 2 Cancer and Leukaemia Epigenetics and Biology Program, Josep Carreras Leukaemia Research Institute (IJC), Campus Can Ruti, Badalona, Spain; 3 PhD Programme in Biomedicine, University of Barcelona, Spain; 4 St. Anna Children’s Cancer Research Institute (CCRI), Vienna, Austria; 5 Germans Trias i Pujol Research Institute (IGTP), Badalona, Spain

## Abstract

Epigenetic regulation occurs on the level of compacting DNA into chromatin. The functional unit of chromatin is the nucleosome, which consists of DNA wrapped around a core of histone proteins. While canonical histone proteins are incorporated into chromatin through a replication-coupled process, structural variants of histones, commonly named histone variants, are deposited into chromatin in a replication-independent manner. Specific chaperones and chromatin remodelers mediate the locus-specific deposition of histone variants. Although histone variants comprise one of the least understood layers of epigenetic regulation, it has been proposed that they play an essential role in directly regulating gene expression in health and disease. Here, we review the emerging evidence suggesting that histone variants have a role at different stages of hematopoiesis, with a particular focus on the histone variants H2A, H3, and H1. Moreover, we discuss the current knowledge on how the dysregulation of histone variants can contribute to hematopoietic malignancies.

## INTRODUCTION

**Figure 1.**
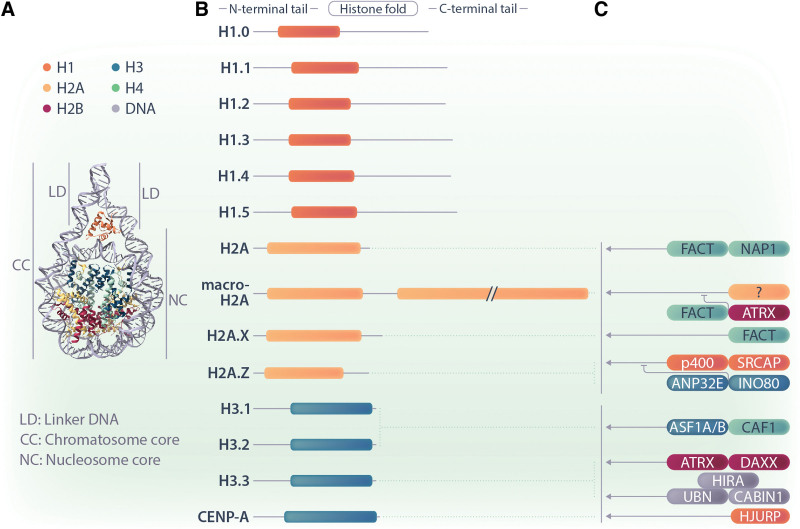
**The diverse families of H1, H2A, and H3 histone variants and their deposition and eviction machineries.** (A) The nucleosome is the structural unit of chromatin. The scheme indicates the position of H2A (yellow), H3 (blue), and H1 (orange) histones. (B) A simplified domain structure of ubiquitously expressed variants for H1, H2A, and H3 are shown. H2A variants differ most strongly in their C-termini, whereas H1 and H3 variants have minimal differences in their domain structures. (C) Some chaperones and ATP-dependent chromatin remodelers are known to specifically deposit or evict histone variants. Their simplified protein names are given, and they are illustrated to reflect their respective interactions with specific histone variants. Regular arrows represent chaperones that are involved in the deposition of histone proteins. The curved inhibitory arrows represent chaperones that are involved in the eviction and/or antagonizm of histone proteins.

In the eukaryotic nucleus, the 3-dimensional compaction of DNA is manifested in the nucleoprotein complex called chromatin. The fundamental functional unit of chromatin is the nucleosome, which consists of 145–147 base pairs of DNA wrapped around an octameric core complex of highly conserved histone proteins. The histone octamer is assembled from a pair of the histone proteins H2A, H2B, H3, and H4 in a replication-coupled manner.^1,2^ A higher-order structure of the nucleosome is achieved by the linker histone H1, which binds the nucleosome core from the outside, and the DNA emerging from it (Figure 1A).^3^ The nucleosome directly controls the accessibility of DNA, and therefore regulates the fine-tuning of gene expression.^4^

Histone proteins can be modified with a variety of posttranslational modifications (PTMs). As these modifications alter chromatin structure, they are critical for the regulation of gene expression programs in all cells. However, any dysregulation of these processes can affect gene expression and the robustness of differentiation programs. Hence, epigenetic alterations affecting nucleosome structure are frequently found in human cancers. This dysregulation can occur through perturbations in multiple mechanisms. These include mutations in genes encoding chromatin regulators and their over or underexpression.^5^ Indeed, malignant cells of various, if not all, types of cancer acquire epigenetic alterations at a high frequency, which has been proposed to aid them in establishing stable oncogenic traits that are required for malignant transformation.^6^ Here, we aim to discuss how epigenetic alterations involving histone variants, their regulators, and effectors might contribute to hematological malignancies.

### Epigenetic changes in hematological malignancies

Epigenetic perturbations in cancer include dysregulated DNA methylation, alterations in chromatin composition, and abnormal patterns of PTMs on histone tails.^7^ Indeed, in hematological malignancies, numerous frequently mutated genes belong to gene families encoding proteins involved in the epigenetic machinery. A prominent example of this is the polycomb-associated gene *ASXL1*, which is found to be recurrently mutated in various hematological malignancies including clonal hematopoiesis, myelodysplastic syndromes (MDS), acute myeloid leukemia (AML), and chronic myelomonocytic leukemia (CMML).^8–10^ *ASXL1* mutations are associated with inferior overall survival.^11,12^ Furthermore, in some cases, mutations in chromatin regulators can co-occur. For instance, co-occurrence of *DNMT3A* and *TET2* mutations is observed at a high frequency in T-cell lymphomas.^13^ Moreover, certain oncogenic fusion proteins, such as PML::RARα, have DNA-binding capacity and are able to aberrantly recruit chromatin regulators and alter gene expression driving leukemogenesis.^14,15^ Alterations to the epigenetic machinery are considered in latest risk-scoring systems for MDS and myeloproliferative neoplasms.^16,17^

Given the high degree of epigenetic dysregulation in hematological malignancies, pharmacological targeting of chromatin regulators, frequently referred to as epigenetic therapies, has attracted significant interest in recent years. For example, the nucleoside cytidine analogue Azacytidine acts as a hypomethylating agent by inhibiting DNA methyltransferases, and is used in the clinic to treat patients with high-risk MDS and AML.^18,19^ A recent report showed that the Menin inhibitor Revumenib efficiently induced remissions in pediatric and adult AML patients with *KMT2A* rearrangements or *NPM1* mutations.^20^ These results stand out as they represent the first efficient epigenetic therapy that is based on the eviction of protein complexes from chromatin. However, not all patients respond to epigenetic therapies, and responses to such therapies are frequently transient. For example, clinical trials involving the preclinically promising DOT1L inhibitor Pinometostat have shown very limited clinical benefit in both children^21^ and adult^22^ patients, and clinical testing was discontinued. Thus, efforts toward the development of more efficient intervention strategies targeting the epigenetic machinery, as well as the combination of these agents with other targeting approaches are needed and currently ongoing.

### Histone variants

An important part of epigenetic regulation in cells occurs on the level of the nucleosome, involving PTMs of histone tails^23^ and the replacement of replication-coupled histones by histone variants.^24^ The replacement of replication-coupled histones by histone variants is still one of the least understood epigenetic mechanisms. Replication-coupled histones H2A, H2B, H3, and H4 are highly conserved among vertebrates, and are encoded by multicopy gene clusters that lack introns.^25^ Synchronized expression during S-phase provides messenger RNAs (mRNAs) of replication-coupled histones lacking poly-A tails for the synthesis of large amounts of histones that are required to efficiently package the newly replicated genome.^26^ In contrast, genes encoding histone variants resemble regular protein-coding genes, and are transcribed by RNA polymerase II (RNA Pol II), expressed throughout the cell cycle, contain introns, and in some cases, feature several splice variants. Histone variants share a histone-fold domain with replication-coupled histones, but otherwise differ in sequence and structure with high variability in their N- and C-termini.^23^ This bestows on histone variants the ability to interact with distinct chaperones and chromatin remodeling complexes, contributing to their locus-specific deposition, which will be discussed in more detail below.

Nucleosomes generally provide a barrier for transcription, limiting transcription factor binding and RNA Pol II elongation. Histone variants affect transcription by altering the dynamics and biophysical properties of nucleosomes. Nucleosome-destabilizing histone variants facilitate gene expression, while nucleosome-stabilizing histone variants exert the opposite effect. The same is true for all other biological processes requiring access to the DNA template, such as DNA repair and replication. Additional complexity and regulatory capacity is introduced through the fact that histone variants can have specific binding partners and specific PTMs.^27,28^

While many and diverse variants exist for histones H2A and H3, very few variants for H2B and H4 have been identified, with minimal variation and restricted expression patterns (Figure 1B). This suggests that a higher evolutionary pressure induced a greater molecular diversification of histones H2A and H3.^29^ Several special features of H2A and H3 exist that could provide an explanation for this phenomenon. First, H2As and H3s engage in more contacts within the nucleosome, sharing interfaces with both their own dimers and other histone proteins. Second, both the N-terminus of H3 and the C-terminus of H2A are close to the DNA entry and exit site of the nucleosome, respectively, and this surface needs to be accessed by most factors involved in epigenetic regulation.^30^

**Figure 2.**
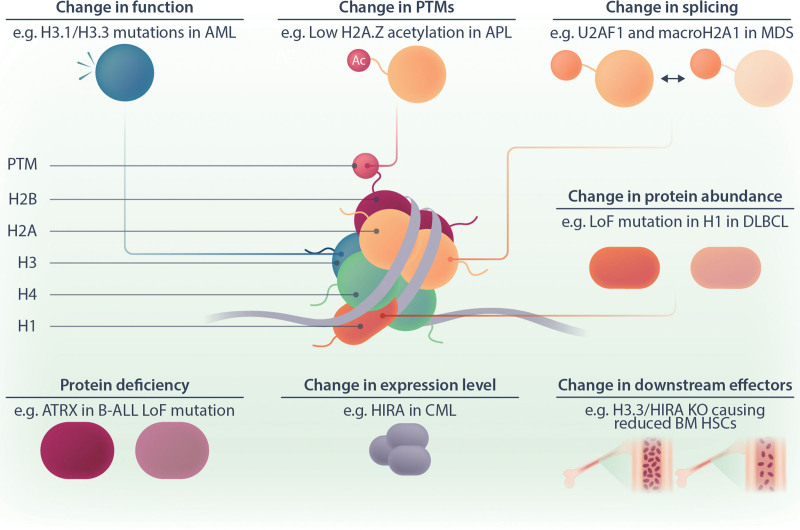
**Dysregulation of histone variants in hematological malignancies.** The cartoon illustrates how histone variants can be dysregulated on several levels. Examples of different types of dysregulation are given. For a more comprehensive list, please see Table 2. AML = acute myeloid leukemia; APL = acute promyelocytic leukemia; B-ALL = B-cell acute lymphoblastic leukemia; BM = bone marrow; CML = chronic myeloid leukemia; DLBCL = diffuse large B-cell lymphoma; HSCs = hematopoietic stem cells; LoF = loss of function; MDS = myelodysplastic syndrome; PTM = posttranscriptional modification.

Here, we discuss the current knowledge about the role of H1, H2A, and H3 histone variants in hematopoiesis and hematopoietic malignancies (Tables 1 and 2; Figure 2). For the role of histone variants in other contexts, we refer readers to reviews by the groups of Bernstein on solid cancers,^75^ Banaszynski on molecular aspects and developmental syndromes,^76^ Buschbeck and Hake on cancer, differentiation, and reprogramming,^24^ and Skoultchi and Bai on linker histones.^77^ More detailed information on specific histone variants and their functions can be found in a special review collection edited by Frederic Berger.^78^

**Table 1:** Loss-of-function Phenotypes of Histone Variants in Mice

Histone Variant	Gene Names and Aliases	Systemic Phenotype	Hematopoietic Phenotype	References
H1.2 and H1.4	*HIST1H1C*, *HIST1H1E*	Viable	GC B cells with increased stem-like characteristics	^31,32^
H1.2, H1.3, and H1.4	*HIST1H1C*, *HIST1H1D*, *HIST1H1E*	Embryonic lethal	Vav-CRE: reduced hematopoiesis and lymphocyte maturation	^33,34^
H2A.X	*H2AFX*	Growth retardation, infertility	Immuno-deficient, reduced lymphocyte number, predisposed to T-cell lymphomas	^35,36^
H2A.B	*H2AB2*, *H2AB3*, H2A.Lap1	Male subfertility	None reported	^37^
H2A.Z1	*H2AZ1*, *H2AFZ*	Embryonic lethal	None reported	^38^
MacroH2A1	*MACROH2A1*, *H2AFY*	Viable, mild metabolic phenotypes	Reduced lymphopoietic potential	^39,40^
MacroH2A1 and macroH2A2	*MACROH2A1, H2AFY; MACROH2A2, H2AFY2*	Viable, growth retardation	None reported	^41^
MacroH2A1.1	*MACROH2A1*, *H2AFY*	Viable	Reduced lymphopoietic potential	^42^
H3.3	*H3F3A* and *H3F3B*	Reduced survival	H3.3 KO leads to reduced HSC numbers and biased myelopoiesis, KO activates endogenous retroviruses	^43^
H3.3	*H3F3B*	Semilethal, infertility	None reported	^44^
H3.3	*H3F3A*	Embryonic lethal	None reported	^45^
CENP-A	*CENPA*	Embryonic lethal	None reported	^46^

GC = germinal center; KO = knock out.

**Table 2:** Involvement of Histone Variants and Their Chaperones in Hematological Malignancies

Histone Variant	Experimental Approach and Observation	Study Object	Blood Cancer^*a*^	References
H1	Frequent mutations affecting H1.2, H1.3, H.4, and H1.5 encoded by *HIST1H1B*,*C*, *D*, and *E*	Patient samples	DLBCL, FH	^47–49^
	Knock out of H1.2 and H1.4 increase Bcl2-driven lymphomagenesis	In vivo mouse studies	DLBCL	^32^
	Recurrent mutations in *HIST1H1B*, *D*, *E*	Patient samples	WM	^50^
	Low expression of *HIST1H1* genes associated with good prognosis in NPM+ cases	Patient samples	AML	^51^
H2A.B	Upregulation promotes transcription of ribosomal genes and protein synthesis	Patient samples, cell lines	HL	^52,53^
	Localizes to site of DNA-synthesis, shortens S-phase and increases susceptibility to DNA damage	Cell lines	HL	^54^
H2A.Z	Tip60 acetylates H2A.Z and is required for embryonic and adult HSC maintenance and *HOXA9* expression in MLL::AF9 transformed HSPCs	Murine transplantation model, cells ex vivo	AML	^55,56^
	Affinity chromatography coupled to mass spectrometry shows that H2A.Z binds the atypical retinoid ST1926	Cell line NB-4	APL	^57^
	Genomic-binding sites of AML::ETO and PML::RARa are characterized by low H2A.Z acetylation	Cell lines NB-4 and SKNO-1	AML, APL	^58^
	Reduction and redistribution upon Myc-induced lymphomagenesis, antagonism with DNA methylation	Murine pre-B cells	BL	^59^
MacroH2A	Loss of macroH2A1.1 splice isoform in U2AF1 S34F linked with reduced differentiation capacity	Patient samples	MDS, AML	^39,60,61^
	Reduced expression of *MACROH2A1* when locus lost in del(5q)	Patient samples	MDS	^42^
	Downregulation of *MACROH2A2* causes reduced erythroid differentiation in vitro	Cell line I/11	N/A	^62^
	*MACROH2A1* mRNA and macroH2A2 protein levels are associated with elevated risk and poor prognosis	Patient samples	T-cell lymphoma, AML	^63,64^
	Case with a fusion gene of *MACROH2A1* and *MECOM*	Patient samples	AML	^65^
H3.1	K27M substitution in *HIST1H3H*	Patient samples	AML	^66^
	KO of *CHAF1B* results in depletion of BM HSPCs	In vivo mouse studies	AML	^67^
	ASF1A accelerates CML blast crisis, activates Notch signaling, and enhances differentiation arrest by enhancing H3K56ac.	Cell lines K562 and MEG01	CML	^68^
H3.3	K27M substitution in *H3F3A*, A26P substitution in *H3F3A*	Patient samples	AML, AML secondary to CMML	^66^
	HIRA expression is enhanced in CML and its downregulation results in cell cycle arrest, limiting overall differentiation but inducing differentiation of leukemic cells to megakaryocytes	Patient samples, cell line K562	CML	^69^
	HIRA regulates RUNX1 during early hematopoietic specification	Murine embryonic stem cells	N/A	^70^
	Cre-mediated *Hira* deletion depletes mouse HSCs and induces erythroid differentiation	In vivo mouse studies, murine primary cells	N/A	^71^
	Cre-mediated deletion of *Daxx* in murine HSC causes upregulation of a PU.1 regulated transcriptional program that causes neutrophilia	In vivo mouse studies, murine primary cells	N/A	^72^
	K27M/I amino acid substitution accelerates AML1::ETO-driven leukemia and decreases disease latency	In vivo mouse studies	AML	^73^
	ATRX loss is a driver of malignancy	Patient samples	BCP-ALL	^74^

^*a*^AML = acute myeloid leukemia; APL = acute promyelocytic leukemia; BCP-ALL = B-cell precursor acute lymphoblastic leukemia; BL = Burkitt’s lymphoma; BM = bone marrow; CML = chronic myeloid leukemia; DLBCL = diffuse large B-cell lymphoma; FH = follicular lymphoma; HL = Hodgkin lymphoma; HSPCs = hematopoietic stem and progenitor cells; MDS = myelodysplastic syndrome; WM = Waldenström’s macroglobulinemia.

## THE DIVERSE GROUP OF H2A VARIANTS

H2A variants represent the most diverse group of core histone variants, including H2A.B, H2A.X, H2A.Z, and macroH2A. H2A.X is the most abundant variant, making up ≈10% of the total H2A content of mammalian cells.^24^ Sequence differences in their histone-fold domain, including the L1-loop, directly affect the biophysical and biochemical properties of H2A variant-containing nucleosomes, foremost their stability in the presence of high salt concentrations in vitro. The most variable region in H2A variants is the C-terminus. While it is virtually nonexisting in H2A.B, it features 2 additional domains in macroH2A. H2A C-termini protrude out of the complex structure of the nucleosome near the DNA exit and entry site, and the N-termini of histone H3s. H2A variants affect virtually all biological processes involving the chromatin template. Here, we will largely focus on the role of H2A variants in regulating gene expression, as this role is thought to underlie their function in cancer. For an extended reading, we recommend reviews by the groups of Valdes-Mora on H2A.Z,^79^ Hake on H2A.Z, B, and X,^80^ and Buschbeck and Heidel on macroH2A.^81^ For a discussion of the roles of H2A variants in DNA repair, we refer readers to a recent review by Oberdoerffer and Miller.^82^

### H2A.X guards genome integrity in lymphocytes

H2A.X can be regarded as an intermediate between a replication-coupled histone and a histone variant.^80^ It can be incorporated into chromatin in a replication-coupled manner, which explains its broad genome-wide distribution. The phosphorylation of H2A.X at Ser139 by the kinase ataxia-telangiectasia mutated is an early signal at sites of DNA damage, and frequently referred to as γ-H2A.X.^83^ Additional modifications of H2A.X fine-tune the intensity and length of the DNA repair signal.^28,82^ Further replication-independent deposition of H2A.X at DNA damage sites by the facilitates chromatin transcription (FACT) complex potentiates the DNA damage signal and repair process.^84^ Apart from DNA damage, several studies have reported that H2A.X can affect the transcription of particular sets of genes, both in a positive and negative manner that is still poorly understood.^80^

H2A.X knock out (KO) mice have compromised immune functions due to reduced number of lymphocytes.^35^ These animals are prone to the development of T-cell lymphomas, which is accelerated in the absence of p53 and attributed to increased genomic instability.^36^ Furthermore, γ-H2A.X has been extensively used to assess the induction of DNA damage by various therapeutic agents.^85^ Additionally, it has been recently reported that the phosphorylation state of H2A.X is capable of controlling human stem and progenitor cell fate decisions, and that its pharmacological modulation could help to overcome differentiation blockade in human leukemia.^86^

### H2A.Z is a highly regulated and polyvalent histone variant involved in gene transcription

H2A.Z was reported to have diverse and, to some extent, opposing functions in gene expression. Two independent genes and 1 event of alternative splicing give rise to 3 H2A.Z isoforms.^87–90^ The chromatin remodeling ATPase complexes p400 and SRCAP are responsible for the deposition of H2A.Z into chromatin,^91,92^ while ANP32E and INO80 mediate its removal.^93,94^ In vivo KO studies showed that H2A.Z is essential for early embryonic development in mammals.^38^ Whether H2A.Z is required for hematopoiesis has not been tested in lineage-specific KO studies, but loss of p400 induces severe defects in hematopoietic development.^95^

H2A.Z destabilizes nucleosomes at promoters and enhancers to facilitate transcription. Yet, it was also shown to have an antagonistic role, as it can form repressive and stable nucleosomes (discussed in ref.^79^). The explanation for this paradox lies in the context: the consequence of H2A.Z deposition into chromatin is dependent on its PTMs, the presence of binding partners, and the composition of the nucleosome, and in particular, the presence of other histone variants. For instance, the combination H2A.Z with H3.3 at active promoters and enhancers positively regulates gene expression.^96,97^ Similarly, acetylation of H2A.Z by p300 or Tip60 promotes gene expression.^98,99^

Until now, little is known about a possible role of H2A.Z in blood cancers, and the available evidence is indirect. The acetyltransferase Tip60 is required for embryonic and adult hematopoietic stem cell (HSC) maintenance and mixed lineage leukemia (MLL) fusion-driven leukemia.^55,56^ In both cases, Tip60 functions are associated with H2A.Z acetylation on key effector genes such as *HOXA9* in leukemia. Conversely, in other fusion-protein driven leukemias, binding sites of AML::ETO and PML::RARα are characterized by low H2A.Z acetylation.^58^ One study suggested that H2A.Z could have a higher affinity for atypical retinoids, thus linking DNA damage with histone variants in PML::RARα-positive leukemia cells.^57^ In the context of a lymphoid cancer, a reduction of H2A.Z levels and its extensive genomic redistribution accompanied Myc-driven B-cell transformation.^59^

### H2A.B is a nucleosome destabilizer involved in Hodgkin lymphoma

H2A.B is a short H2A isoform lacking a C-terminal tail. It appeared late in the evolution of eutherian mammals, is fast evolving, and its expression is restricted to the testis.^100^ Incorporation of H2A.B leads to very unstable nucleosomes, thus promoting open chromatin structures and gene expression.^80^ Testis-specific genes, including several of those encoding chromatin regulators, are frequently re-expressed in cancer.^101^ In line with this, H2A.B was found to be upregulated in Hodgkin lymphoma cell lines and primary samples.^52,53^ H2A.B binds proliferating cell nuclear antigen (PCNA) and accelerates DNA replication, thereby leading to increased susceptibility for DNA damage.^54^ In addition, H2A.B promotes protein synthesis by increasing the transcription of ribosomal RNAs and protein-coding mRNAs by RNA Pol I and II , respectively.^52^ As H2A.B is most abundantly expressed in the testis, KO mice display male subfertility associated with abnormal sperm function.^37^ A hematopoietic phenotype in these mice has not been reported.

### Multidomain histone variants with a role in hematopoiesis

MacroH2A is unique among H2A histone variants, as it possesses a 60 amino acid long unstructured C-terminal linker followed by a globular macrodomain. Like H2A.Z, macroH2A exists in 3 forms that are encoded by 2 independent genes, and 1 event of alternative splicing. A splicing switch from macroH2A1.2 to macroH2A1.1 expression is frequently associated with cell differentiation.^102^ In contrast to H2A.B, macroH2A is frequently linked to gene repression^103^ and contributes to the 3-dimensional compaction of repressed heterochromatin.^104^ The mechanisms regulating the genome-wide distribution of macroH2A are not well understood. However, it has been shown that the FACT complex removes macroH2A from transcribed regions, and ATRX is a negative regulator of macroH2A deposition on subtelomeric regions.^105,106^ In terms of biological function, a large number of studies on reprogramming, differentiation, and development have suggested that a major function of macroH2A is to promote and stabilize the differentiated state of cells.^24^

Mice lacking both macroH2A genes are viable, but display reduced pre and postnatal growth, which was attributed to altered expression of metabolic genes in the liver.^41^ While the same study did not observe any phenotype in the hematopoietic system, deletion of the *MACROH2A1* gene alone or only the alternatively-spliced exon encoding macroH2A1.1 caused reduced capacity of HSCs to differentiate into B and T cells in competitive transplantation assays.^39,42^ Interestingly, mutations in the splicing factor U2AF1 occur frequently in MDS, and cause a splice isoform switch from macroH2A1.1 to macroH2A1.2. This is functionally relevant, as reintroduction of macroH2A1.1 was sufficient to partially rescue defects in erythroid and B-cell differentiation in cells and mice bearing mutant U2AF1.^39,60,61^ MacroH2A2 was shown to have a role in erythroid development, as downregulation of macroH2A2 expression caused reduced erythroid differentiation in a cell culture model.^62^

In patients, anecdotal evidence has linked macroH2A to disease etiology and risk. In a single AML case, macroH2A1 has been found as part of a fusion with the *MECOM* gene encoding the transcription factor EVI1.^65^ MacroH2A1 is one of the many genes on chromosome 5q that is frequently lost in MDS.^42^ In T-cell lymphoma and AML, macroH2A1 mRNA and macroH2A2 protein, respectively, are part of signatures that are associated with elevated risk and poor prognosis.^63,64^ Taken together, the knowledge about the involvement of H2A variants in hematopoietic malignancies until now is limited, but point to tumor-promoting roles of H2A.B, while macroH2A1.1 might act as a possible differentiation-promoting tumor suppressor.

## H3 HISTONES HAVE SPECIFIC DEPOSITION MACHINERIES

In mammals, there are 3 main H3 variant classes with a ubiquitous expression pattern. The first class consists of the replication-coupled histones H3.1 and H3.2, termed the canonical H3 histones. H3.1 and H3.2 differ by a single amino acid substitution of Cys96 to Ser96, respectively.^107^ The second class of ubiquitously expressed H3 variants refers to the replication-independent, so-called replacement histone H3.3, which can replace the canonical H3.1 and H3.2 histones at the promoters of active genes, either in a replication-independent or replication-coupled manner. The highly abundant H3.3 variant is encoded by 2 genes, *H3F3A* and *H3F3B*, which give rise to the same protein sequence, but differ in their untranslated regions. H3.3 histone differs from canonical H3.2 by 4 amino acids in positions 31, 87, 89, and 90, and from H3.1 by 5 amino acids, with an additional variation at position 96.^108,109^ Lastly, the third main H3 histone variant centromere protein A (CENP-A) is encoded by *CENPA*, and is specifically deposited to centromeres. CENP-A plays an important role in genome stability through its central involvement in kinetochore assembly.^110^ Human CENP-A shares ≈60% sequence homology with the C-terminal histone-fold domain of replication-coupled H3s, whereas its N-terminal tail is highly unique in comparison to other H3 variants.^111^

The correct deposition, repair, exchange, and removal of H3 variants within the nucleosome is mediated by specific histone chaperones. Replication-coupled chromatin assembly during the S-phase of the cell cycle, where H3.1 and H3.2 variants are incorporated into chromatin, is facilitated by the synergistic action of antisilencing factor 1 A/B (ASF1A/B) and chromatin assembly factor 1 (CAF-1).^112,113^ The characteristic, euchromatin-associated deposition of the replication-independent H3.3 variant in gene bodies, enhancers, and promoters is orchestrated by the HIRA complex, which is composed of histone cell cycle regulator A (HIRA), ubinuclein 1/2 (UBN1/2), and calcineurin-binding protein (CABIN1).^114–116^ H3.3 is also incorporated into telomeric and pericentromeric chromatin regions. This deposition is facilitated by the protein complex formed by α-thalassemia mental retardation syndrome X-linked (ATRX) and death domain-associated protein (DAXX).^114^ Both HIRA and ATRX-DAXX chaperone complexes specifically recognize H3.3, due to the presence of Ala87 and Gly90 substitutions that distinguish this variant from replication-coupled H3s.^115,117^ The recruitment of CENP-A to the nucleosome is facilitated by its specific chaperone Holliday junction recognition protein (HJURP).^118^ The deposition mechanisms for the other H3 variants have not been thoroughly described, although H3.Y.1 and H3.Y.2 variants were observed to interact with the HIRA chaperone complex.^75^ As we will detail below, alterations in either H3 variants or their chaperones are able to adversely influence transcriptional programs, hence have the potential to initiate malignancy-associated gene expression profiles.

### Replication-coupled H3 variants and their chaperones in hematopoiesis and leukemogenesis

Because the canonical H3.1 variant plays an essential role in replication-coupled nucleosome assembly, its correct expression and localization is essential for a healthy gene expression profile. Indeed, profiling of histone mutations in primary human AML samples by next-generation sequencing (NGS) has identified K27M, K27I, and Q69H amino acid substitutions in patients with de novo AML and AML secondary to myelofibrosis, occurring due to mutations in genes encoding H3.1 (*HIST1H3H, HIST1H3F*, and *HIST1H3A*, respectively).^66^ Moreover, the same study has found that H3.1 Q69H and K27M/I mutations alter HSC frequency and differentiation potential in vitro and in vivo. When examined for leukemic potential, it was established that H3.1 K27M/I mutations increase AML cell proliferation and disease aggressiveness.^66^

The chaperone CAF-1 for replication-coupled histone deposition is composed of 3 separate functional subunits, one of which is the p60 subunit named chromatin assembly factor 1B (CHAF1B).^119^ Investigations in *CHAF1B* KO mice revealed that this chaperone subunit is required for normal hematopoiesis, as its loss resulted in the depletion of hematopoietic stem and progenitor cells (HSPCs) in the bone marrow (BM). The same study found that CHAF1B overexpression, however, resulted in much higher proliferation rates of HSCs and a leukemic phenotype. Furthermore, overexpression of *CHAF1B* in MLL::AF9 fusion oncoprotein-expressing cells drastically enhanced leukemia development in mice, while the deletion of this chaperone subunit induced differentiation of MLL::AF9 AML cells.^67^ These results highlight the CAF-1 canonical histone chaperone acting as an important molecular switch, regulating normal hematopoiesis and leukemogenesis. Additionally, ASF1A, the other replication-coupled histone chaperone, was identified as an enhancer of differentiation arrest in chronic myeloid leukemia (CML) cells. Mechanistically, ASF1A was proposed to play an active role in initiating Notch signaling and upregulating H3K56 acetylation, which in turn resulted in CML acceleration and blast crisis.^68^ Roles of H3.2 and CENP-A in regulating hematopoiesis or driving hematological malignancy have not yet been described in literature. Taken together, the above-mentioned studies suggest that H3.1 and its chaperones play a role in the maintenance of healthy hematopoiesis, and perturbations to their healthy state can drive or accelerate leukemias.

### H3.3 and its chaperones regulate hematopoietic homeostasis

Several studies have identified the H3.3 variant and its chaperones to play a role in the fine-tuned regulation of hematopoiesis. Recently, Guo et al^43^ reported that the double KO of the mouse orthologs of *H3F3A* and *H3F3B* resulted in reduced numbers of long-term HSCs, together with the expression of a granulocyte/monocyte progenitor-like transcriptional signature, increased bias toward the myeloid lineage over lymphopoiesis, splenomegaly, and a loss of terminally differentiated erythroid cells. The authors, thus, concluded that H3.3 maintains HSC stemness and restrains their myeloid bias. A complementary study accounted for the requirement for the H3.3-specific chaperone *Hira* in adult mouse HSCs self-renewal, and for the incorporation of H3.3 at genomic regions that are enriched for binding sites of hematopoietic transcription factors to restrain erythroid differentiation.^71^ Additionally, in mouse embryonic stem cells (mESCs), Hira was identified to interact with Runx1, a transcription factor (TF) that is essential for the early hematopoietic specification from hemogenic endothelium.^120^ Through this interaction, Hira was shown to modulate hematopoiesis by regulating *Runx1* expression and localization through enhancing the enrichment of H3.3 within the intronic enhancer of *Runx1*.^70^ Moreover, mice with Cre-mediated deletion of *Hira* in HSCs present with reduced numbers of BM HSCs, anemia, thrombocytopenia, and lymphocytopenia. Further analyses in this mouse model demonstrated that Hira is required for the expression of TFs and signaling molecules that are essential for HSC development and maintenance, and that Hira establishes a HSC-specific DNA accessibility signature, including Spib/PU.1 sites.^121^ Indeed, Cre-mediated KO of the other H3.3-specific chaperone Daxx in mice further revealed that loss of *Daxx* changes the global genomic distribution of H3.3, as well as histone modifications in hematopoietic progenitors. These conditional KO mice showed an activation of PU.1-dependent transcriptional programs with biased myelopoiesis, causing neutrophilia and inflammation,^72^ both of which are crucial drivers of oncogenesis.^122^

### Perturbations in H3.3 and its chaperones as a driver of hematological malignancies

In addition to their roles in the regulation of normal hematopoiesis, the H3.3 variant and its chaperones have been found to be implicated in hematological malignancies. For instance, Boileau et al^66^ have identified K27M and A26P mutations in the H3.3 variant-encoding *H3F3A* gene in patients with secondary AML and AML secondary to CMML. A study investigating the role of H3 mutations in leukemic transformation showed that the H3.3 K27M/I mutations drastically accelerated AML1::ETO-driven AML and decreased disease latency. However, this was not observed in mice with MLL::AF9-driven AML, hinting at context-dependent effects of H3.3 mutants in leukemic transformation.^73^ Additionally, expression of *HIRA* was found to be upregulated in the CML cell line K562 and in the BM of patients with CML; and its downregulation in K562 cells induced cell cycle arrest, reduced proliferation, as well as differentiation into megakaryocytes.^69^ Finally, deep NGS of cancer-associated genes in samples from pediatric acute lymphoblastic leukemia (ALL) patients identified *ATRX* as a novel cancer driver gene in B-cell precursor ALL patients.^74^ Taken together, the H3.3 variant and its chaperones stand as an interesting candidate for further exploration of their roles in leukemogenesis and the regulation of normal hematopoiesis.

## DEFICIENCY OF DIVERGENT H1 LINKER HISTONES IN LYMPHOMA

The interaction of the more evolutionary divergent H1 linker histones with chromatin occurs outside the core nucleosome. Of the 7 somatic H1 variants that are present in mammals, H1.1 to H1.5 are replication-dependent, while H1.0 and H1.X are replication-independent.^123^ All H1 histones share the same overall structure consisting of a short N-terminus, a globular domain responsible for chromatin loading, and an unstructured C-terminal linker domain mediating interactions with effector proteins.^77^ The redundant function of all H1 linker histones is chromatin compaction and reducing DNA accessibility.^124–126^ Both overexpression and depletion of H1 genes reduces cell survival and proliferation, suggesting that a correct dosage of H1 is important.^33,127^ In addition to these redundant functions, several studies have suggested isoform-specific functions for some H1 variants (further discussed in ref.^123^), which might be related to differences in their PTMs and binding partners, such as the polycomb-repressive complex 2.^128^

Mice can tolerate global deletion of the genes encoding H1.2 and H1.4, but additional deletion of H1.3 causes embryonic lethality.^31,34^ Mice with hematopoietic lineage-specific codeletion of H1.2 H1.3 and H1.4 are viable, but the consequential 50% reduction of H1 protein levels negatively affects hematopoiesis.^33^ Specifically, these mice have fewer HSPCs in the BM and lower numbers of mature B and T lymphocytes in peripheral lymphoid tissues. H1 genes are commonly mutated in follicular lymphoma, Waldenström’s macroglobulinemia, and diffuse large B-cell lymphoma (DLBCL) in a manner that is predicted to be a loss-of-function, and reduce the total dosage of H1 proteins.^32,47–50^ The potential relevance of this observation has been recently evaluated in a study showing that germinal center B cells of H1.2 and H1.4-deficient mice re-express stemness genes. When crossed with a transgenic DLBCL mouse model of *Bcl2* overexpression, H1 deficiency aggravated the disease.^32^ This suggests that a reduction in the levels of H1 contributes to lymphomagenesis.

## CONCLUSIONS

The contribution of chromatin regulation and epigenetics to the regulation of hematopoiesis and driving of hematological malignancies is only partially understood. Current research focuses on exploring, and if possible, exploiting the less understood chromatin regulatory pathways. Emerging evidence suggests that replacement of replication-coupled histones with replication-independent histone variants fine-tunes regulation of hematopoiesis. Consequently, deregulation of histone variants has been implicated in leukemia and lymphoma. It is important to note that, histone variants can have both tumor suppressive and tumor-promoting functions indicating a strong context-dependence. For instance, H1 variants present with loss-of-function mutations in lymphoid malignancies, whereas gain of function mutations are prevalent in H3.1 and H3.3 in myeloid malignancies. Reports of mutations in genes encoding other histone variants remain anecdotal.

More work is needed to clarify the scenarios in which histone variants may provide a point for therapeutic intervention. As histone variants are structural proteins and difficult to target with small compounds, potential intervention strategies might aim at targeting their upstream regulators or downstream effectors. Potentially, investigating any associations between driver mutations as well as the subsequent malignant transcriptional programs in hematological malignancies and histone variant-associated chromatin signatures may provide a window of therapeutic intervention. However, to this date, our knowledge about chaperones and chromatin remodelers that deposit histone variants is incomplete, and even less is known about the molecular pathways that are upstream and downstream of these factors. Thus, further studies are required to assess whether approaches that involve targeting of histone variants could assist in improving clinical outcomes in hematological malignancies. Another gap in our knowledge is whether histone variants contribute to the noncell autonomous regulation of hematopoiesis and hematopoietic disorders such as by influencing the function of the stem cell niche in the BM microenvironment. All in all, histone variants are an exciting aspect of epigenetic regulation, and we have only started to gain insight into their role in hematopoiesis and hematopoietic malignancies.

## AUTHOR CONTRIBUTIONS

All authors were involved in conceptualizing, writing, reviewing, and editing the article.

## DISCLOSURES

The Buschbeck lab receives funding from CellCentric Ltd for research unrelated to the topic of this review. The authors have no conflicts of interest to disclose.

## SOURCES OF FUNDING

Research in the Buschbeck lab is supported by the following grants: the Marie Skłodowska Curie Training network “INTERCEPT-MDS” H2020-MSCA-ITN-2020-953407, the national grant PID2021-126907NB-I00 from FEDER/Ministerio de Ciencia e Innovación (MCIN) - Agencia Estatal de Investigación, AGAUR 2021-SGR-260, the Fundació La Marató de TV3 257/C/2019 and the Fundación AECC PRYGN222668BUSC. OM is funded by the predoctoral MCIN fellowship PRE2019-088529. Research at the IJC is supported by the “La Caixa” Foundation, the Fundació Internacional Josep Carreras and the CERCA Programme/Generalitat de Catalunya. Research in the Grebien lab is supported by the Marie Skłodowska Curie Training network “INTERCEPT-MDS” H2020-MSCA-ITN-2020-953407 and the Austrian Science Fund (projects P-35628, P-35298 and TAI-490).
